# Isolated Pubic Ramus Fractures Are Serious Adverse Events for Elderly Persons: An Observational Study on 138 Patients with Fragility Fractures of the Pelvis Type I (FFP Type I)

**DOI:** 10.3390/jcm9082498

**Published:** 2020-08-03

**Authors:** Pol Maria Rommens, Johannes Christof Hopf, Michiel Herteleer, Benjamin Devlieger, Alexander Hofmann, Daniel Wagner

**Affiliations:** 1 Department of Orthopedics and Traumatology, University Medical Center Mainz, Langenbeckstrasse 1, 55131 Mainz, Germany; johannes.hopf@unimedizin-mainz.de (J.C.H.); michiel.herteleer@unimedizin-mainz.de (M.H.); benjamin.devlieger@unimedizin-mainz.de (B.D.); daniel.wagner@unimedizin-mainz.de (D.W.); 2 Department of Orthopedics and Traumatology, Westpfalz Klinikum Kaiserslautern, Hellmut-Hartert Straße 1, 67655 Kaiserslautern, Germany; Hofmann.trauma-surgery@gmx.net

**Keywords:** pubic ramus fractures, fragility fractures of the pelvis, mortality, living condition, mobility, quality of life

## Abstract

Background: Fractures of the pubic ramus without involvement of the posterior pelvic ring represent a minority of fragility fractures of the pelvis (FFP). The natural history of patients suffering this FFP Type I has not been described so far. Material and methods: All patients, who were admitted with isolated pubic ramus fractures between 2007 and mid-2018, have been reviewed. Epidemiologic data, comorbidities, in-hospital complications, and one-year mortality were recorded. Of all surviving patients, living condition before the fracture and at follow-up was noted. Mobility was scored with the Parker Mobility Score, quality of life with the European Quality of Life 5 Dimensions 3 Level (EQ-5D-3L), subjective sensation of pain with the Numeric Rating Scale (NRS). Results: A consecutive series of 138 patients was included in the study. There were 117 women (84.8%) and 21 men (15.2%). Mean age was 80.6 years (SD 8.6 years). 89.1% of patients presented with comorbidities, 81.2% of them had cardiovascular diseases. Five patients (4%) died during hospital-stay. Median in-hospital stay was eight days (2–45 days). There were in-hospital complications in 16.5%, urinary tract infections, and pneumonia being the most frequent. One-year mortality was 16.7%. Reference values for the normal population of the same age are 5.9% for men and 4.0% for women. One-year mortality rate was 22.2% in the patient group of 80 years or above and 8.8% in the patient group below the age of 80. The rate of surviving patients living at home with or without assistance dropped from 80.5% to 65.3%. The median EQ-5D-Index Value was 0.62 (0.04–1; IQR 0.5–0.78). Reference value for the normal population is 0.78. Average PMS was 4 and NRS 3. Within a two-year period, additional fragility fractures occurred in 21.2% and antiresorptive medication was taken by only 45.2% of patients. Conclusion. Pubic ramus fractures without involvement of the posterior pelvis (FFP Type I) are serious adverse events for elderly persons. During follow-up, there is an excess mortality, a loss of independence, a restricted mobility, and a decreased quality of life. Pubic ramus fractures are indicators for the need to optimize the patient’s general condition.

## 1. Introduction

Pubic ramus fractures are part of complex pelvic injuries in high-energy trauma [1]. They also occur in isolation or in combination with fractures of the posterior pelvis due to low-energy trauma. In communities with a high life expectancy, the incidence of fragility fractures of the pelvis (FFP) surpasses that of high-energy pelvic trauma [2,3]. Of all FFP, isolated pubic rami fractures represent only one fifth [4]. They are described as FFP Type I in the classification of Rommens and Hofmann [4]. A combination of fractures of the pubic ramus with fractures of the posterior pelvic ring is present in more than 80% of FFP. These fracture combinations are described as FFP Types II, III, or IV in the same classification [4]. Pubic ramus fractures are usually diagnosed on a conventional pelvic radiograph, which is the first diagnostic step after a domestic fall with pain in the pelvic region. On this radiograph, crush zones and fissures or non-displaced fractures in the sacrum may be overlooked. The real extent of the pelvic injury is then underestimated. With a pelvic CT, fractures and dislocations of the posterior pelvis can be analyzed in more detail [5]. Consequently, a pelvic CT is indispensable for complete evaluation of an FFP. Multiple publications deal with epidemiology, treatment, and outcome of elderly patients with pubic ramus fractures [6,7,8,9,10,11]. None of them differentiates between isolated pubic ramus fractures and pubic ramus fractures in combination with posterior pelvis fractures. In this retrospective analysis, we first analyzed the pelvic CT-images of all patients admitted with FFP in an 11.5-year period. We then looked at quality of life, mobility, level of independence, and mortality of patients with FFP Type I (isolated pubic ramus fractures) under exclusion of the FFP Types II, III, and IV. We compared our data with available data on the level of independence of the same patients before the fall and with data on quality of life and mortality of a representative group in the normal population.

## 2. Patients and Methods

**Figure 1:**
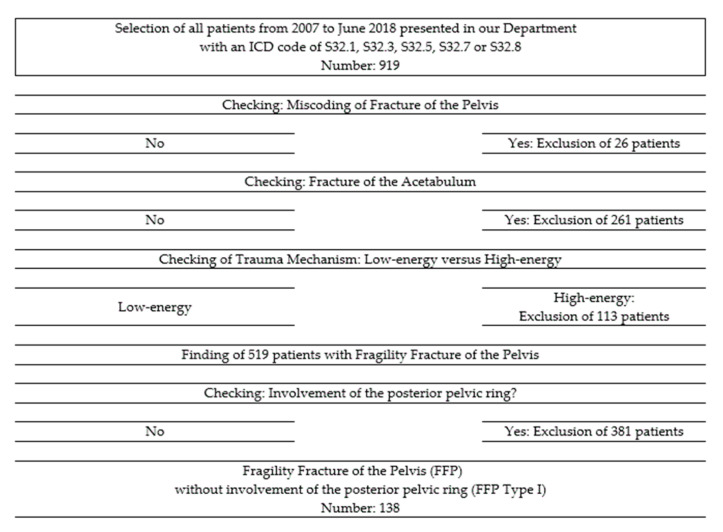
Flowchart of selected and excluded patients.

We retrospectively reviewed the medical charts and radiological data (pelvic overviews and pelvic CT-images) of all patients with an FFP, who presented at the Department of Orthopedics and Traumatology of the University Medical Center Mainz, Germany between 1 January 2007 and 30 June 2018 (11.5-years period). We identified 519 patients with this diagnosis. We analyzed the pelvic CT-data of these patients to identify involvement of the posterior pelvic ring and classified all FFP according to the classification of Rommens and Hofmann [4]. We then excluded FFP with a non-displaced or displaced fracture of the posterior pelvic ring (FFP Type II (*n* = 251), Type III, and Type IV (*n* = 130)). We also excluded patients with acetabular fractures, patients with pelvic fractures due to high-energy trauma and patients with pelvic fractures due to malignancies. In addition, 138 of 519 (26.6%) of FFP were classified as FFP Type I. A flowchart of selected and excluded patients is presented in Figure 1. They build the patient cohort for this study.

The following epidemiologic data of these 138 patients were collected: age, sex, trauma mechanism, conservative or operative treatment, duration of hospital stay, in-hospital complications and destination at discharge from hospital. The patient’s records were searched for the physical status just before admission, current medication and for the following comorbidities at the time of first presentation: cardiovascular disease, pulmonary disease, osteoporosis, malignancies, diabetes mellitus, rheumatoid arthritis, and dementia. Images were assessed for existing hip replacement or lumbar spine fusion. In-hospital medical complications included cardiovascular events, pneumonia, symptomatic deep venous thrombosis, symptomatic pulmonary embolism, symptomatic urinary tract infection, and skin ulcer. 

Patients or their relatives were contacted by phone asking to answer several questionnaires. If this was not possible, their general practitioner or the bureau of vital statistics was contacted to ask about vital status. Quality of life was assessed by a European Quality of Life five dimensions—three level (EQ-5D-3L) questionnaire, and scores range from 0 to 1.0 (higher scores indicating better quality of life) [12,13,14]. Subjective sensation of pain was rated with the numeric rating scale (NRS), with scores from 0 to 10 (higher scores indicating heavier pain) [15]. The actual place of living and mobility status was asked, the mobility further specified by Parker Mobility Score (PMS), ranging from 0 to 9, higher scores equal to better mobility [16]. Patients were asked for the occurrence of subsequent fragility fractures. 

Mean values with standard deviation (SD) were given in case of normal distribution, median values with min and max together with 25–75 inter quartal ranges (IQR) in case of non-normal distribution. Continuous data were compared using the unpaired Mann–Whitney U test for non-normally distributed data. Linear regression was tested with Spearman Rho. The chi-square test was used to compare nominal groups. A *p*-value of ≤ 0.05 was considered statistically significant. Statistical analysis was performed using SPSS software (IBM SPSS Statistics for Windows, Version 23.0; IBM Corp, Armonk, NY, USA).

## 3. Results

### 3.1. Baseline Characteristics

In addition, 138 patients with fragility fractures of the anterior pelvic ring only (FFP Type I) were identified. There were 130 patients with unilateral pubic rami fractures (FFP Type Ia = 94.2%) and eight patients with bilateral fractures (FFP Type Ib = 5.8%). Mean body mass index (BMI) of all patients was 24 (15–42). Sex ratio was 5.6/1 in favor of women. The average age of women was 5.6 years higher than that of men. The vast majority of patients suffered a fall from standing position or recurrent falls. Only 15 patients (10.9%) had no comorbidities at first presentation. Among the patients with comorbidities (89.1%), cardiovascular diseases, osteoporosis, and actual or previous malignancies were the most frequently present. In addition, 107 patients received anticoagulants and/or antithrombotic drugs (77.5%). Median time delay between trauma and primary presentation was 0 days (0–264, IQR 0–2). More than 84% of patients presented within one week after trauma. Patients with a presentation of seven or more days after trauma were younger (77.7 years ± 8.14 vs. 81.3 years ± 8.64, *p* = 0.045), had shorter stays in hospital (median 4 vs. 8 days, *p* < 0.001) and they suffered medical complications less often (3.8% vs. 22.3%, *p* = 0.030).

Demographics, trauma mechanism, time of presentation, comorbidities, relevant medication, and previous surgeries at hip joint or lumbar spine of all patients, prior to suffering an FFP Type I, are depicted in Table 1.

Age and sex of patients with pubic ramus fractures, published in representative original articles of the last decades are listed in Table 2

### 3.2. Care Pathways

In total, 136 patients were treated conservatively (98.6%). Conservative treatment consisted of pain therapy, mobilization in bed, sitting and standing at the bedside, followed by short transfers and assisted walking. Weight bearing was allowed at all times, when tolerated. Two patients were treated operatively (1.4%). In one patient, a retrograde transpubic screw was inserted and another patient received a plate and screw osteosynthesis of the anterior pelvic ring.

Fourteen patients (10.1%) received an outpatient treatment, and 124 patients were admitted in our trauma unit (89.9%). Five patients died during hospital stay (4.0%). The reasons of death were not directly related to the pubic ramus fractures: pneumonia in two, cardiovascular arrest, cardiac infarction and multiple organ failure in one patient each. Median in-hospital stay of the surviving patients was eight days (2–45 days, IQR 5–10 days). Twenty-two patients (16.5%) suffered complications during hospital stay of which urinary tract infection and pneumonia were the most frequent. Thrombosis and pulmonary embolism were not noticed. Complications occurred more often with a longer stay in hospital (median 11 days vs. 7 days, *p* <0.001) and in patients with more comorbidities (median 2 vs. 1, *p* = 0.085). They did not differ with age (median 83.5 years vs. 81 years, *p* = 0.201). At discharge, almost three quarters of the patients were mobile on the ward or in the room. Nearly two thirds of patients could be discharged at home, and the remaining were discharged in different caring institutes. More data on in-hospital complications, on mobility, and destination at discharge are depicted in Table 3.

### 3.3. Follow-Ups

**Figure 2:**
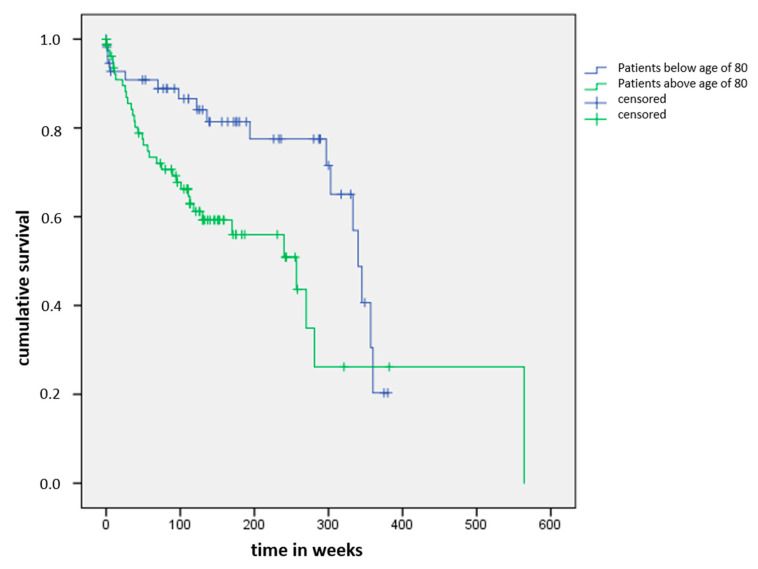
Kaplan–Meier curve of the cumulative survival of patient groups below and above age of 80.

Forty-five patients had died at follow-up (32.6%). Median delay between primary presentation and death was 50 weeks (0–345 weeks, IQR 22–112 weeks). Twenty-three patients died within the first year after primary presentation (16.7%), 41 within 5 years (29.7%). None of the patients died due to a direct complication of the pubic ramus fractures. One-year mortality of patients below and above 80 years was 8.8% resp. 22.2%. Five-year mortality of patients below and above 80 years was 17.5% resp. 39.5%. Figure 2 shows the Kaplan–Meier curve of the cumulative survival of both age groups.

One-year mortality rates of reference populations and of published series of patients with FFP are listed in Table 4.

Seventeen patients were lost to follow-up (12.3%) and 4 refused participation in our retrospective study (2.9%). In addition, 72 of 93 surviving patients completed the questionnaires (77.4%). Median time delay between presentation and follow-up of the responding 72 patients was 149 weeks (53–382 weeks, IQR 113–237 weeks). All patients had a minimum follow-up of one year. At follow-up, the vast majority of patients was able to walk independently or with walking aids. The rate of surviving patients living at home with or without assistance dropped from 80.5% to 65.3%. Detailed data on mortality, mobility, and living conditions before trauma and at follow-up is depicted in Table 5.

The median Parker Mobility Score was 4 (0–9; IQR 3–7). The median EQ-5D-Index Value of 72 patients, who answered this questionnaire, was 0.62 (0.04–1; IQR 0.5–0.78). The median value of NRS was 3 (0–10, IQR 0–5). The EQ-5D-Index Value correlated with the PMS (*p* < 0.001), inversely with age (*p* < 0.001) and with NRS (*p* = 0.011). The number of comorbidities correlated inversely with the EQ-5D-Index Value (*p* = 0.022) and with the PMS (*p* = 0.010). The occurrence of a medical complication did not influence the quality of life (EQ-5D-Index Value) at follow-up (*p* = 0.107). Nevertheless, the PMS was higher in patients without medical complication (median 4 vs. 3, *p* = 0.072). There was no correlation of the EQ-5D-Index Value with delayed presentation (*p* = 0.529).

Fourteen out of 66 patients (21.2%), who answered this question, expressed having suffered an additional fragility fracture after the pubic ramus fractures: five times a progression of the FFP Type I to another FFP Type, three times an osteoporotic vertebral fracture, three times a fracture of the lower extremities, two times rib fractures, and once a fracture of the upper extremities. The median time delay between the index injury and the new fracture was 123 weeks (46–413, IQR 58–167).

Of 66 patients, who answered this question, only 28 (45.2%) expressed taking an anti-resorptive medication.

## 4. Discussion

This is the first and unique study on mobility, level of independency, quality of life, and survival rate of elderly patients with isolated pubic ramus fractures. Isolated pubic ramus fractures are the least unstable fracture types in FFP [4]. Stability of the pelvic ring with pubic ramus fractures and intact peri-pelvic soft tissues is only reduced by 7% [25]. It seems reasonable to hypothesize that isolated pubic ramus fractures have limited impact on the patient’s outcome and can be treated with “careful neglect”. The results of this study show the opposite: isolated pubic ramus fractures are serious adverse events for elderly persons as they have a distinct influence on their mobility, level of independence, and survival rate.

Age and sex ratio of our patient cohort was similar to that of the populations of previous publications, although all types of FFP were included in previously published studies.

The mean age of the patients in the presented papers ranges from 75.3 to 83 years, sex ratio was always in favor of women and ranged from 2.2/1 to 8/1. The variation in sex ratio can be explained by the different inclusion criteria used (e.g., minimum age). Demographic data of our 138 patients—mean age of 80.6 years and female to male ratio of 5.6/1—are situated in the middle of this spectrum of data.

Unilateral pubic ramus fractures were diagnosed in 94.2% and bilateral in 5.8%. These findings are in accordance with data from other publications: Hill et al. found bilateral fractures in 3.5% in a series of 286 consecutive patients [7], and Krappinger et al. in 6.6% in a series of 534 patients [8].

Only 10.9% of our patients presented without comorbidities. The very high rate of comorbidities is a direct sign for the vulnerability of this patient population. Hypotensive drugs, impaired vision, vertigo, walking impairment and intake of analgesics may be at the origin of their falls [26,27,28]. In a multidisciplinary approach, the reasons for the falls must be explored and treated, if possible. Multidisciplinary co-management prevents recurrent falls and further deterioration of the patient’s condition [29].

Complication rate within hospital-stay was 16.5%, urinary tract infection and pneumonia being the most frequent complications. For comparison, van Dyck et al. registered 20.2% of in-hospital complications, of which urinary tract infection and pneumonia also were the most frequent [18]. Banierink et al. calculated a complication rate of 23% within 30 days after injury, in which delirium was included, whereas the last was not represented in other studies [20]. In-hospital complications occurred more often in patients with a longer hospital stay and with more comorbidities. It is therefore most important for reducing the length of hospital stay while providing uninterrupted medical care and continuous support for mobilization.

One-year mortality of our patients was three times higher than in the reference population. One-year mortality rate of our male patients was 19.1%, of our female patients 16.2%. The one-year mortality rates in the reference population are 5.9% for males and 4% for females [24].

In the studies listed in Table 4, there always is an excess mortality in FFP patients when compared with the general population of the same age, be it in Scotland, the Netherlands, Austria, or Germany. As in the publications of Krappinger [8] and Banierink [20], we found a strong dependence of the one-year mortality on age: the rates of our patients below and above the age of 80 were 8.8% resp. 22.2%. In both groups below and above the age of 80, mortality rates exceeded the rates of the general population. We did not find a remarkable difference in the one-year mortality between our study population without involvement of the posterior pelvic ring and other study populations, in which at least a part had involvement in the posterior pelvic ring. The one-year mortality rate of 16.7% in our patient cohort is situated at the lower end of data given in the published series in Table 4, ranging from 11.2% [8] to 26.8% [20].

More than three quarters of surviving patients could be reached for follow-up. We registered a clear reduction of the level of independence at follow-up. The percentage of patients living independent at home dropped from 61.1% to 37.5%, and the rate of patients living in a nursing home increased from 8.3% to 19.4%. The median PMS of our patients was 4. PMS rates mobility at home (3 points), mobility out of home (3 points), and shopping (3 points). The score has a minimum of 0 (immobility) and a maximum of 9 (independent mobility). Our patients scored low, which means that most of them needed help in their activities of daily life. These data are also supported by the EQ-5D-Index Value of our patients being 0.62. This value is 0.78 for a previously described German patient population older than 75 years of age [30]. Unsurprisingly, there was a significant positive correlation between quality of life (EQ-5D-Index Value) and mobility (PMS). Patients with more comorbidities had a lower quality of life and were less mobile. With these data, it becomes clear that isolated pubic ramus fractures cannot be regarded merely as “tissue damage”. The results of our study show a similarity instead between the natural course of patients with an FFP Type I and patients with more unstable types of FFP, as shown in Table 4. As the origin of the fragility fractures can be multifactorial, a multidisciplinary co-management is needed for the treatment of existing comorbidities [29].

Patients scored on average 3 points in their subjective sensation of pain, depicted in the NRS. NRS scores range from 0 (no pain) to 10 (unbearable pain) [15]. A value of three points means that patients suffer from a lower degree of continuous burden in their daily life, even after a follow-up of at least one year.

One fifth of our patients suffered an additional fragility fracture during follow-up, whereas only 45.2% took anti-resorptive medication. The additional fractures do not occur immediately but rather later during follow-up, in our series after a median of 123 weeks. van Dyck published a rate of 24.1% additional fractures of which the majority occurred in the first 24 months [18]. Maier et al. found that only 50% of their 93 patients received anti-osteoporotic therapy [19]. Suhm et al. stated that only 46% of their 50 follow-up patients received any form of anti-resorptive therapy and additional fractures occurred in 22% [31]. Although the occurrence of isolated pubic rami fractures is a serious adverse event for elderly persons, prevention of further fractures with anti-resorptive drugs seems not to be widely accepted. One important part of geriatric co-management is exploration of bone metabolism and treatment of osteoporosis for the prevention of the occurrence of additional fragility fractures [29,31].

This study has several limitations. It concerns a retrospective study, in which patients were recruited over a time of more than 10 years. The follow-up time varied from one to more than seven years. The influence of pubic ramus fractures on mobility, level of independency, and quality of life may become less important with greater time interval. We could not compare our data with other series of patients with FFP Type I, as they are not available in literature. We compared our results with those of patient series, which also involved fractures of the posterior pelvic ring and found several similarities. The published series so far are not completely comparable with our patient group, but—to the best of our knowledge—represent the most comparable data available. From our data, we cannot give recommendations for a different, more specific type of conservative treatment of the fractures themselves. Our data rather show that pubic ramus fractures arise as the consequence of co-existing problems of different origin. These problems should be explored and treated by multidisciplinary geriatric co-management [29].

## 5. Conclusions

Pubic ramus fractures without involvement of the posterior pelvic ring are serious adverse events for elderly persons. They are much more than merely a tissue damage, which can be treated with careful neglect. They have a direct influence on level of independence, mobility, and quality of life of these patients. One-year mortality is three times as high as in a reference population. Pubic ramus fractures are a warning signal of the patient to his/her medical caretakers and simultaneously a demand for exploration and treatment of comorbidities, which may lead to recurrent falls, additional fragility fractures, and further deterioration of his/her general condition.

## Figures and Tables

**Table 1:** Baseline characteristics of all patients before suffering an FFP Type I.

	Number	%	Mean Age	SD
**Demographics**				
All patients	138	100	80.6	8.6
Women	117	84.8	81.5	8.2
Men	21	15.2	75.9	9.2
Patients younger than 80	57	41.3	72.5	6.1
Patients age 80 and older	81	58.7	86.2	4.8
**Trauma Mechanism**	**Number**		**%**	
Fall from standing position	106		77	
Other trauma mechanism	19		13.8	
Recurrent falls	7		5.1	
No trauma memorable	6		4.2	
**Time of Presentation**	**Number**		**%**	
Day of trauma	86		62.3	
Within first week	30		21.7	
After one week	22		16.0	
**Comorbidities**	**Number**		**%**	
Patients without comorbidities	15		10.9	
Patients with comorbidities	123		89.1	
Cardiovascular disease	112		81.2	
Osteoporosis	54		39.1	
Actual/previous malignancies	28		20.3	
Diabetes mellitus	27		19.6	
Pulmonary disease	7		12.3	
Dementia	15		10.9	
Rheumatoid arthritis	5		3.6	
**Medication**	**Number**		**%**	
Anticoagulants	107		77.5	
Cortisone	20		14.5	
**Previous surgeries**	**Number**		**%**	
Total hip arthroplasty	20		14.5	
Lumbar spine fusion	5		3.6	

**Table 2:** Age and sex of patients with pubic ramus fractures in previous publications.

Author [Reference]	Year	Number of Patients	Age (Range)	Sex (F/M
Hill et al. [7]	2001	286	74.7 (17–97)	231/55
Taillander et al. [17]	2003	60	83 (65–99)	54/6
Krappinger et al. [8]	2009	534	73.3 (SD 19.8)	448/86
van Dyck et al. [18]	2010	99	80.1 (60–98)	88/11
Maier et al. [19]	2016	93	76.8 (n.a.)	64/29
Banierink et al. [20]	2019	153	79 (65–100)	108/45
Schmitz et al. [21]	2019	196	75.3 (60–94)	138/58
Hamilton et al. [10]	2019	43	78.4 (S.D 9.2)	32/11
Loggers et al. [11]	2019	117	83 (65–97)	101/16
Own series	2020	138	80.6 (52–98)	117/21

**Table 3:** In-hospital complications, mobility, and destination at discharge.

	Number	%
**In-Hospital Complications**		
All in-hospital patients	124	100
In-hospital death	5	4.0
Number of patients with in-hospital complications	22	17.7
**Type of In-Hospital Complication**		
Urinary tract infection	17	13.7
Pneumonia	7	5.6
Skin ulcer	6	4.8
Cardiovascular accident	3	2.4
**Mobility at Discharge**		
All surviving in-hospital patients	119	100
Type		
Mobile on the ward	68	57.1
Mobile in the room	18	15.2
Need help for out-of-bed mobilization	15	12.6
Immobilized in bed	0	0
Unknown	18	15.2
**Destination of Discharge**		
All surviving in-hospital patients	119	100
Type		
Discharge at home	74	62.2
Geriatric rehabilitation center	13	11.0
Geriatric clinic	10	8.4
Nursing home	10	8.4
Short-time nursing care	6	5.0
Assisted living environment	3	2.5
Other hospital	1	0.8
Unknown	16	13.4

**Table 4:** One-year mortality rates of reference populations and of published series of patients with FFP.

Author (Reference)	Year	Number	Mean Age of Patients	One-Year Mortality Rate, in %	Reference Group in %
Hill et al. [7]	2001	286	74.7	13.3	4.7 ***
Leung et al. [22]	2001	60	78	11.7	
Krappinger et al. [8]	2009	534	73.3	11.2	
Krappinger et al. [8]	2009	270 *	n.a.	3.7	
Krappinger et al. [8]	2009	264 **	n.a.	18.9	12.5 ****
van Dyck et al. [18]	2010	99	80.1	24.7	7.5 *****
Maier et al. [19]	2016	93	76.8		
Andrich et al. [23]	2017	5685	80	21	11
Banierink et al. [20]	2019	153	79	26.8	
Banierink et al. [20]	2019	65	76–8531	5 *****	
Banierink et al. [20]	2019	34	>85	35	15 *****
Loggers et al. [11]	2019	117	83	23	
Loggers et al. [11]	2019	94	n.a.	20 ^Ɨ^	
Own series	2020	138	80.6	16.7	5.9 ^¥^/4.0 ^¥¥^
Own series	2020	57 *	74	8.8	
Own series	2020	81 **	86	22.2	
Own series	2020	21 ^ƭ^	75	19.1	5.5
Own series	2020	117 ^ƭ^	82	16.2	4.0

* Patients below age of 80, ** Patients with age of 80 and above, *** Data from the Scottish Institute for Statistics, **** Data from Austrian Institute for Statistics, ***** Data from the Dutch Institute for Statistics, ^Ɨ^ Patients with isolated anterior pelvic ring fractures, ^¥^ Male population [24], ^¥¥^ Female population [24].

**Table 5:** Mortality, mobility and living condition before trauma and at follow-up.

	Number		%	
All patients	138		100	
Death at follow-up	45		32.6	
Lost to follow-up	17		12.3	
Refused participation	4		2.9	
Remaining patients	72		52.2	
**Mobility at Follow-Up**				
All patients	72		100	
Type				
Independent walking	20		27.8	
Walking with walking aid	36		50	
Walking inside home	12		16.7	
Immobilized in bed	4		5.6	
**Living Condition at Primary Presentation and at Follow-Up**				
	**Primary Presentation**		**Follow-Up**	
	**Number**	**%**	**Number**	**%**
Number of patients	72	100	72	100
Independent at home	44	61.1	27	37.5
At home with assistance	14	19.4	20	27.8
Nursing home	6	8.3	14	19.4
Hospitalized	3	4.2	1	1.4
Assisted living environment	2	2.8	4	5.6
Unknown	3	4.2	6	8.3
